# A Novel Two-Component Signaling System Facilitates Uropathogenic *Escherichia coli*'s Ability to Exploit Abundant Host Metabolites

**DOI:** 10.1371/journal.ppat.1003428

**Published:** 2013-06-27

**Authors:** Wentong Cai, Yvonne Wannemuehler, Giuseppe Dell'Anna, Bryon Nicholson, Nicolle L. Barbieri, Subhashinie Kariyawasam, Yaping Feng, Catherine M. Logue, Lisa K. Nolan, Ganwu Li

**Affiliations:** 1 Department of Veterinary Microbiology and Preventive Medicine, College of Veterinary Medicine, Iowa State University, Ames, Iowa, United States of America; 2 Laboratory Animal Resources, College of Veterinary Medicine, Iowa State University, Ames, Iowa, United States of America; 3 Departamento de Biofísica, Universidade Federal do Rio Grande do Sul, Porto Alegre, Rio Grande do Sul, Brasil; 4 Department of Veterinary and Biomedical Sciences, Pennsylvania State University, University Park, Pennsylvania, United States of America; 5 Laurence H. Baker Center for Bioinformatics and Biological Statistics, Iowa State University, Ames, Iowa, United States of America; University of Utah, United States of America

## Abstract

Two-component signaling systems (TCSs) are major mechanisms by which bacteria adapt to environmental conditions. It follows then that TCSs would play important roles in the adaptation of pathogenic bacteria to host environments. However, no pathogen-associated TCS has been identified in uropathogenic *Escherichia coli* (UPEC). Here, we identified a novel TCS, which we termed KguS/KguR (KguS: α-ketoglutarate utilization sensor; KguR: α-ketoglutarate utilization regulator) in UPEC CFT073, a strain isolated from human pyelonephritis. *kguS*/*kguR* was strongly associated with UPEC but was found only rarely among other *E. coli* including commensal and intestinal pathogenic strains. An *in vivo* competition assay in a mouse UTI model showed that deletion of *kguS*/*kguR* in UPEC CFT073 resulted in a significant reduction in its colonization of the bladders and kidneys of mice, suggesting that KguS/KguR contributed to UPEC fitness *in vivo*. Comparative proteomics identified the target gene products of KguS/KguR, and sequence analysis showed that TCS KguS/KguR and its targeted-genes, *c5032* to *c5039*, are encoded on a genomic island, which is not present in intestinal pathogenic *E. coli*. Expression of the target genes was induced by α-ketoglutarate (α-KG). These genes were further shown to be involved in utilization of α-KG as a sole carbon source under anaerobic conditions. KguS/KguR contributed to the regulation of the target genes with the direct regulation by KguR verified using an electrophoretic mobility shift assay. In addition, oxygen deficiency positively modulated expression of *kguS*/*kguR* and its target genes. Taken altogether, this study describes the first UPEC-associated TCS that functions in controlling the utilization of α-ketoglutarate *in vivo* thereby facilitating UPEC adaptation to life inside the urinary tract.

## Author Summary

Successful colonization requires bacterial pathogens to adapt their metabolism to the conditions encountered in particular infection sites. Two-component signaling systems (TCSs) enable bacterial pathogens to sense and respond to environmental cues, thus mediating their adaptation to environmental change. Though many TCSs that have been characterized in commensal *E. coli* have been associated with UPEC pathogenesis, no characterized TCS has been significantly associated with UPEC strains. Here, we characterized a UPEC-associated TCS that was localized to a genomic island. This novel TCS and its target genes were involved in anaerobic utilization of α-ketoglutarate, an abundant metabolite in UPEC infection site-renal proximal tubules, thus contributed to UPEC fitness *in vivo*. Our results also suggest that this TCS controls a variant tricarboxylic acid (TCA) cycle, the first described in *E. coli*, which links the oxidative and reducing branches under anaerobic conditions. Similar TCA branches have been identified in other bacterial pathogens that have adapted to oxygen-limited, obligate anaerobic conditions, and/or intracellular carbon sources. Therefore, this study provides new insight into the adaptation of bacterial pathogens to nutrient availability *in vivo* and makes possible the discovery of targets for antimicrobial treatment.

## Introduction

Urinary tract infection (UTI) is one of the most common bacterial infections in humans and is a significant clinical issue worldwide. Annually, UTIs are associated with 7 million office visits, 1 million emergency room visits, 100,000 hospitalizations, and $1.6 billion in healthcare costs [1]. In fact, ∼80–90% of community-acquired UTIs are caused by uropathogenic *Escherichia coli* (UPEC) [2]. Many virulence factors are required for UPEC to cause UTIs. Typically, UTIs begin with urethral contamination with UPEC from the bowel [3]. Successful attachment to the uroepithelium requires specific adhesins including P, type 1 and other fimbriae (such as F1C, S, M, and Dr fimbriae) [4], [5]. UPEC may then ascend the urethra to enter the bladder and kidneys, where several highly regulated virulence factors, including fimbriae, secreted toxins (hemolysin, Vat, Sat, and CNF) and polysaccharide capsule, may contribute to colonization and pathogenesis [6]. It is likely that UPEC's ability to colonize the urinary tract and cause disease is affected by its adaptive responses to local environmental cues, including changes in nutrient availability.

Urine is an important host environment that UPEC encounter after transition from the intestine to urinary tract. From the standpoint view of *E. coli* nutrition, urine is a mixture of amino acids and small peptides in low concentrations, with the notable exception of urea, which is abundant [7]. UPEC adapt to growth in urine by using peptides and amino acids as their primary carbon source for fitness [8]. About 85% of UPEC strains retain a complete *dsdCXA* locus [9] and these genes are responsible for the detoxification of D-serine allowing UPEC to use D-serine as the sole carbon and nitrogen source in urine [9]. Iron is another vital nutrient for UPEC but free iron concentrations in the mammalian host are extremely low. Consequently, UPEC have evolved multiple systems and strategies to obtain needed iron during infection including *ent*, *iro*, *chu*, *sit*, *iutA* and *fyuA* [10]–[13]. In addition, the urinary tract is considered to be a nucleotide-deficient environment. Study of *pyrD* and *guaA* deletion mutants showed that metabolism of nucleobases is required for UPEC colonization of the bladder [14], [15]. Thus, UPEC metabolism appears to play a critical role in colonization and invasion of the host. Therefore, it is not surprising that acquisition of genomic islands encoding metabolic pathways is often essential for the colonization of host niches. Gene cluster *ttrABC* and *ttrRS*, responsible for utilizing tetrathionate as an electron acceptor in the intestine, are encoded on *Salmonella enterica* subsp. *enterica* serotype Typhimurium pathogenicity island II and contribute to detoxification of tetrathionate and confer a competitive advantage in growth [16]–[18]. Gene *nixA* within a transferable genomic island in *Helicobacter pylori* aids in transport of nickel and consequently results in enhanced activity of urease, a key factor for fitness and virulence [19]–[22]. Besides islands encoding iron uptake systems, no other metabolic island has yet been characterized in UPEC even though their study would help understand the pathobiology and facilitate the identification of potential targets for novel therapeutic approaches to prevent this prevalent pathogen.

The pathways, conferring metabolic adaptation of bacterial pathogens to the host milieu, are usually controlled by both global and specific regulators. One mechanism used by most bacterial pathogens to sense and respond to nutrient availability is two-component signaling systems (TCSs). TCSs, composed of a membrane-bound sensor histidine kinase (HK) and a cytoplasmic response regulator (RR), have been implicated in regulating the response of bacteria to a wide array of signals and stimuli, including nutrients, quorum signals, antibiotics, etc. The recognition of physical or chemical signals by HK sensor domain typically triggers the modulation of its autophosphorylation activity. The phosphoryl group is then transferred to the RR, usually a DNA binding protein that functions by altering gene expression [23], [24]. In *E. coli* K12, more than 30 TCSs have been identified and characterized to some extent [25]–[27]. Of these, several were shown to be involved in UPEC pathogenesis. TCS BarA/UvrY controlled efficient switching between glycolytic and gluconeogenic carbon sources and contributed to UPEC virulence [28]. Deletion of QseC, the kinase of a well-known TCS QseC/QseB, dysregulated nucleotide, amino acid, and carbon metabolism and resulted in attenuation of UPEC virulence [29], [30]. In addition, PhoQ/PhoP [31] and the AirS system [32] have been linked to UPEC pathogenesis. To date, all TCSs linked to UPEC pathogenesis have been common to *E. coli* and are not pathogen-associated. Given the distinct features of environments colonized by UPEC, we hypothesize that UPEC may possess special TCSs that sense urinary tract-specific metabolic signal(s) and adapt UPEC metabolism to available nutrients.

Here, we describe the first TCS significantly associated with UPEC and its direct target genes encoded on a small genomic island. In addition, we demonstrate that the expression of these island genes is induced in response to α-KG, a nutrient found in high concentration in renal proximal tubule cells, and that these genes are involved in the utilization of α-KG under anaerobic conditions. Our results suggest that KguS/KguR contributes to UPEC fitness *in vivo* by facilitating the utilization of a host abundant metabolite α-KG. This study provides a new perspective for understanding UPEC pathogenesis *in vivo*.

## Results

### Identification of a novel TCS in UPEC CFT073

**Figure 1:**
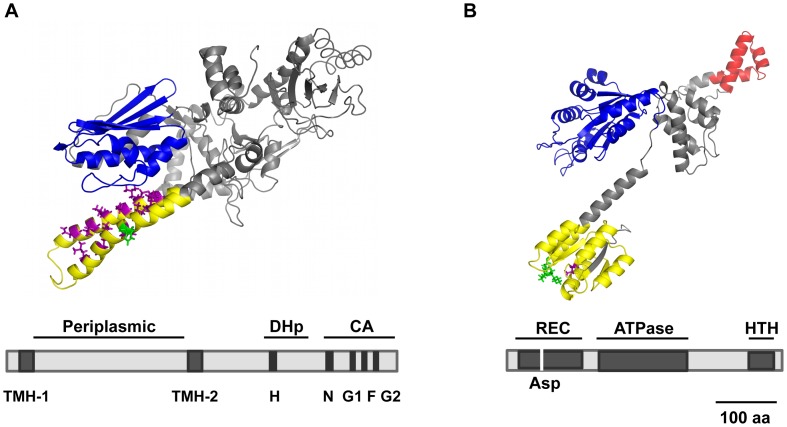
Predicted 3-D structures and conserved domains of the HK C5041 and the RR C5040. The exact positions of each domain are described in the text. 3-D structures were predicted by I-TASSER online server. (A) HK C5041. The signature segments (H, N, G1, F, and G2) of the kinase domain (CA, blue) and dimerization and histidine phosphorylation domain (DHp, yellow and purple) were identified by sequence alignment and indicated. The conserved histidine residue is indicated by green. The two transmembrane helices (TMH-1 and TMH-2) were predicted by transmembrane protein topology prediction tool TMMOD. (B) RR C5040. The receiver domain (yellow) containing the putative aspartate residue (Asp, purple) for phosphorylation during signaling cascade, the AAA^+^ ATPase domain (blue), and the helix-turn-helix (HTH, red) domain for DNA binding are shown.

TCSs enable bacteria to sense, respond, and adapt to a wide range of environments, stressors, and growth conditions [23]. Since UPEC encounter unique environmental conditions found within their host, as compared to commensal *E. coli* and diarrheagenic *E. coli*, we hypothesize that specific TCSs may exist and facilitate UPEC adaptation to certain host niches. Indeed, analysis of the UPEC CFT073 genome identified a putative TCS *c5041*/*c5040* by the presence of specific domains in their respective predicted proteins (Fig. 1). Domains and three-dimensional structures of C5041 and C5040 were predicted by the threading method using the I-TASSER online server (http://zhanglab.ccmb.med.umich.edu/I-TASSER/) and subsequently refined using four-body contact potentials. C5041 is predicted to be a typical sensor kinase harboring a dimerization and histidine phosphotransfer (DHp) domain and a histidine kinase-like ATPase domain (Fig. 1A). The C-terminal 288 amino acids of C5041 contains five short signature segments, H-, N-, G1-, F-, and G2-boxes, which are conserved across most histidine kinases [33], [34]. The histidine residue within the H-box, which is very likely phosphorylated during the signaling process, was found at position 394. In the N-terminal section of C5041, two transmembrane helices were identified at position 15–35 and at position 297–317, respectively. Between the two transmemebrane helices, a periplasmic domain, which might serve as a signal sensor, was identified according to the distribution of charged residues ahead and behind the postulated transmembrane helices. The periplasmic domain of C5041 is 25.3% identical to that of DctB, a sensor kinase protein for dicarboxylates transport in *Sinorhizobium meliloti* (of which crystal structure is available) [35], suggesting C5041 may sense analogs of succinate, which is sensed by DctB [36].

C5040 is 37% identical to the nitrogen regulatory protein C (NtrC) [37], [38] at the amino acid sequence level. C5040 harbors the CheY-like receiver domain, phosphorylation site, AAA^+^ ATPase domain, and helix-turn-helix (HTH) domain (Fig. 1B). The receiver domain (position 13–123) catalyzes phosphoryl transfer from the HK to itself and regulates the output. The aspartate residue, which is predicted to be phosphorylated in the signal transduction cascade, was found at position 58. In the C-terminal part of C5040, a helix-turn-helix domain was identified, which is a DNA-binding domain that recognizes enhancer-like sequences. The CheY-like receiver domain, AAA^+^ ATPase domain, and helix-turn-helix (HTH) domains of C5040 are 32.5%, 51.7%, and 41.7% identical to that of NtrC, respectively. Such characteristics are consistent with our expectations for a TCS. Additionally, DNA sequence analyses were performed using the search engine at http://blast.ncbi.nlm.nih.gov/Blast.cgi and *c5041* and *c5040* did not show any homology to any characterized histidine kinase or response regulator genes, suggesting that C5041/C5040 is a novel TCS.

### TCS genes *c5041* and *c5040* are significantly associated with UPEC and increase CFT073 fitness *in vivo*

Sequence analysis of all *E. coli* genomes available online showed that *c5041*/*c5040* and its orthologs are absent from commensal and diarrheagenic *E. coli*, but present in UPEC strains. Also, genotyping of 317 *E. coli* clinical isolates including human diarrheagenic *E. coli* and UPEC (Table S3) for *c5041* and *c5040* revealed their differential distribution. Both genes of this TCS were amplified in 139 of 200 UPEC (69.5%) but were not detected in 12 enterotoxigenic *E. coli*, 66 enterohemorrhagic *E. coli* or 39 enteropathogenic *E. coli* isolates. Therefore, *c5041*/*c5040* appears to be significantly associated with UPEC, raising the possibility that C5041/C5040 contributes to UPEC fitness or virulence *in vivo*.

**Figure 2:**
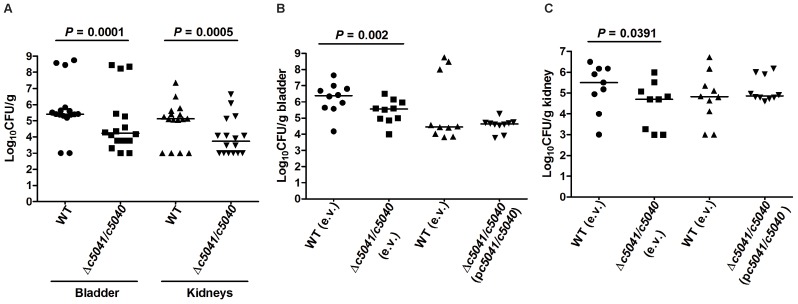
Deletion of *c5041*/*c5040* reduces the colonization of the murine urinary tract by UPEC strain CFT073. (A) The WT and Δ*c5041*/*c5040*::Chl^r^ mutant strains were mixed to a 1∶1 ratio and approximately 2×10^9^ CFU were transurethrally inoculated into female mice. Two days after infection, the mice were sacrificed and their bladders and kidneys were aseptically removed. WT and mutant bacteria were recovered by plating homogenized tissue samples on LB or LB containing chloramphenicol and their viable counts were determined. (B) and (C), for the *in vivo* complementation assay, a mixture of wild-type strain containing empty vector (e.v.) pGEN-MCS and mutant strain containing empty vector or wild-type strain containing empty vector and mutant strain containing complementation plasmid (p*c5041*/*c5040*) were incolulated to female mice as in (A). Homogenized tissue samples (B, bladder; C, kidneys) were plated on LB with ampicillin or LB with both ampicillin and chloramphenicol. Each dot represents log_10_CFU/g in the bladder or kidney from an individual animal and the detection limit is 1000 CFU/g. Bars indicate the median log_10_CFU/g. A two-tailed Wilcoxon matched pairs test was performed, and the difference in colonization levels of WT and mutants was considered statistically significant if P<0.05.

To study the role of C5041/C5040 in UPEC fitness *in vivo*, we compared the UPEC CFT073 wild-type strain with its Δ*c5041*/*c5040* double deletion mutant strain for the ability to colonize the mouse urinary tract at 48 hours post-inoculation using an *in vivo* competition assay. The double deletion mutation was constructed by replacement of *c5041*/*c5040* with the *cat* cassette conferring chloramphenicol resistance. Though Δ*c5041*/*c5040* mutants grew as well as the wild type (WT) in LB, the mutant was significantly outcompeted by the WT, with a nearly 10-fold reduction in the median CFU/g from both bladders and kidneys at 48 hours post-inoculation (Fig. 2A, *P*<0.05 for both organs). To verify that the impact on colonization is not due to a secondary mutation, *in vivo* complementation experiments were performed. A stable low-copy plasmid pGEN-MCS was used as it was shown to be well maintained in CFT073 up to 48 h even in the absence of antibiotic pressure [39]. Coding regions of *c5041* and *c5040* plus their predicted promoter region were cloned into pGEN-MCS, and the resultant construct was named p*c5041*/*c5040*. As shown in Fig. 2B and C, the Δ*c5041*/*c5040* mutant containing the empty vector (pGEN-MCS) demonstrated an expected colonization defect in bladder and kidneys colonization as compared to the WT (empty vector) (*P*<0.05) while mutants harboring the complementation plasmid (p*c5041*/*c5040*) were able to colonize the bladder and kidneys at wild-type levels at 48 hours post-inoculation. These results clearly demonstrate that *c5041*/*c5040* has a role in UPEC colonization and fitness *in vivo*.

### Proteomic determination of gene products targeted by C5041/C5040

To determine how C5041/C5040 affects UPEC fitness *in vivo*, 2D-DIGE was used to identify differentially expressed gene products in the proteomes of the WT and single deletion mutants of *c5041* and *c5040* grown aerobically in human urine. Human urine was used as the growth medium to simulate the urinary tract environment [40]–[42]. *c5041* and *c5040* single mutants were constructed by replacement with Chl^r^ gene, and the resultant mutants were named LMP100Chl (CFT073 Δ*c5041*::Chl^r^) and LMP101Chl (CFT073 Δ*c5040*::Chl^r^). Using a cutoff of 1.5-fold change, seventeen proteins in the mutants were differentially expressed, as compared to the WT (Fig. S1). Of these, five were induced and five were repressed in Δ*c5041*::Chl^r^ mutant; while six were induced and five were repressed in the Δ*c5040*::Chl^r^ mutant. To determine the identities of the differentially expressed proteins, protein spots in the gel were excised and subjected to enzymatic digestion with trypsin followed by tandem mass spectroscopy. Functions of these proteins primarily fell into three categories: metabolism, cell envelope constituents, and translational machinery (Table 1). C5035, which showed a 24.6-fold induction, is a putative 2-oxoglutarate dehydrogenase presumably involved in tricarboxylic acid (TCA) cycle. XylA, a xylose isomerase, was increased 8.7-fold. It catalyzes the interconversion between xylose and xylulose. SitA, induced 4-fold, is a putative periplasmic iron-binding protein. Several outer membrane proteins including OmpA, NmpC, OmpF and OmpX were uniformly induced in Δ*c5040*::Chl^r^ mutant. 50S ribosomal proteins, such as Rpll, RplQ, and RplF, were also differentially expressed in the mutants as compared to the WT. These results indicate that C5041/C5040 regulate multiple genes, suggesting that they may serve as a pleiotropic two-component system.

**Table 1:** Differentially expressed proteins identified in mutant strains of UPEC CFT073 as compared to the wild-type when cultured in human urine.

Order	Name or ORF	Function	MW^a^/pI^b^	No. of peptides matched	Total ion score	Total ion C.I.^c^ %	Fold-Change^d^
	*Differentially expressed proteins in Δc5041*::*Chl^r^* mutant						
1	C5035	putative 2-oxoglutarate dehydrogenase	49832/6.1	16	172	100	24.6
2	XylA	xylose isomerase	49634/5.63	16	63	100	8.7
3	SitA	putative periplasmic iron-binding protein	31544/8.7	12	284	100	4.0
4	TufB	EF-Tu	43427/5.3	7	556	>95	2.2
5	GlnA	glutamine synthetase	52099/5.26	2	76	>95	2.0
6	YbiS	hypothetical protein	33059/5.99	10	232	100	−1.9
7	SpeE	spermidine synthase	32286/5.33	7	228	100	−2.2
8	RplF	50S ribosomal protein L6	17763/9.49	9	208	100	−2.2
9	RplQ	50S ribosomal protein L17	14355/11.05	6	115	100	−3.2
10	RplI	50S ribosomal protein L9	15759/6.17	14	474	100	−4.0
	*Differentially expressed proteins in Δc5040*::*Chl^r^* mutant						
1	OmpA	outer membrane protein A precursor	41028/6.24	17	449	100	4.2
2	OmpA	outer membrane protein A precursor	41028/6.24	14	317	100	3.0
3	NmpC	outer membrane porin protein nmpC precursor	40277/4.64	6	391	>95	2.1
4	OmpF	outer membrane protein F precursor	39309/4.76	9	496	>95	1.9
5	OmpX	outer membrane protein X precursor	18648/6.56	2	152	>95	1.9
6	RplF	50S ribosomal protein L6	18949/9.71	7	356	>95	1.8
7	RplD	50S ribosomal protein L4	22073/9.72	5	380	>95	−1.9
8	FocD	F1C fimbrial usher	96379/6.72	1	29	>95	−1.9
9	Tig	Trigger protein	48163/4.83	4	150	>95	−2.0
10	TufB	EF-Tu	43427/5.3	11	677	>95	−2.3
11	RplQ	50S ribosomal protein L17	14355/11.05	5	211	100	−4.3

a Molecular weight.

b Isoelectric point.

c Confidence interval (Ion Confidence Interval % greater than 95 were considered significant).

d Average from two biological repeats.

### Genomic island genes targeted by C5041/C5040 contribute to UPEC fitness *in vivo*

**Figure 3:**
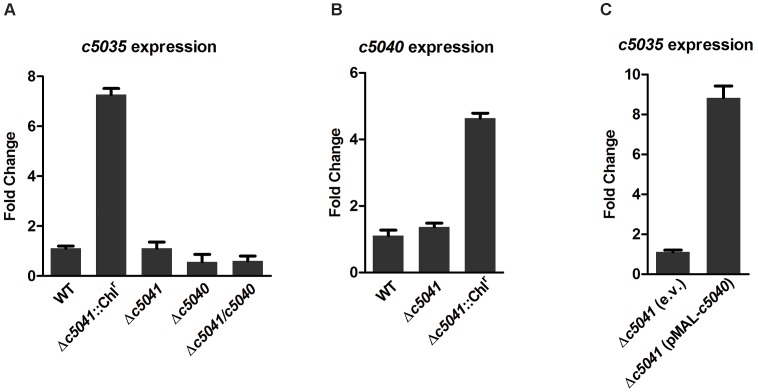
Overexpression of *c5040* upregulated *c5035* expression independent of the natural stimulus. Bacteria were grown in human urine under aerobic conditions before being subjected to RNA extraction. qRT-PCR expression values are means plus standard deviations (error bars) of three independent experiments. (A) *c5035* expression in various TCS mutants compared to WT. (B) *c5040* expression in Δ*c5041* and Δ*c5041*::Chl^r^ mutants compared to WT. (C) *c5035* expression in Δ*c5041* mutant carrying the pMAL plasmid with *c5040* under the control of P_tac_ promoter as compared to Δ*c5041* mutant carrying the empty vector (e.v.). IPTG is present at 1 mM for induction.

The most differentially expressed protein identified by proteomic analysis was C5035, which was induced nearly 25-fold in the Δ*c5041*::Chl^r^ mutant. qRT-PCR results confirmed that *c5035* was up-regulated in Δ*c5041*::Chl^r^ mutant, but not in Δ*c5040*::Chl^r^ mutant, as compared to the WT when grown aerobically in human urine (Fig. 3A). To rule out the possibility of polar effects, the Chl^r^ gene was removed from both Δ*c5041*::Chl^r^ and Δ*c5040*::Chl^r^ mutants to generate mutants LMP100 (CFT073 Δ*c5041*) and LMP101 (CFT073 Δ*c5040*), respectively. Interestingly, the expression of *c5035* was detected at extremely low levels not only in the mutant strains Δ*c5041* and Δ*c5040*, but also in the WT. No significant differences in the *c5035* expression were detected among the WT, Δ*c5041* and Δ*c5040* mutants when grown in human urine under aerobic conditions (Fig. 3A). Note that in the Δ*c5041*::Chl^r^ mutant, *c5041* was replaced by the Chl^r^ gene, which transcribes in the same orientation as its downstream gene, *c5040*. We further compared the expression levels of RR *c5040* in the WT, Δ*c5041*, and Δ*c5041*::Chl^r^ mutants. The results showed that expression of *c5040* was extremely low in the WT and Δ*c5041* mutant, but it was significantly upregulated in the Δ*c5041*::Chl^r^ mutant when grown in human urine under aerobic conditions (Fig. 3B). Therefore, we suspect that the upregulation of *c5035* in Δ*c5041*::Chl^r^ mutant was due to the constitutive expression of *c5040* caused by the upstream Chl^r^ gene. If so, overexpression of *c5040* in the Δ*c5041* mutant should induce the expression of *c5035*. Thus, *c5040* was cloned into plasmid pMAL-MCS under the control of promoter P_tac_ and the resultant plasmid transformed into the Δ*c5041* mutant. Indeed, induction of *c5040* significantly upregulated the expression of *c5035* in the Δ*c5041* mutant (Fig. 3C). We also tested such regulation under different culturing condition and the results revealed similar regulatory pattern. In summary, constitutive expression of *c5040* induced the expression of *c5035* regardless of growth conditions.

**Figure 4:**
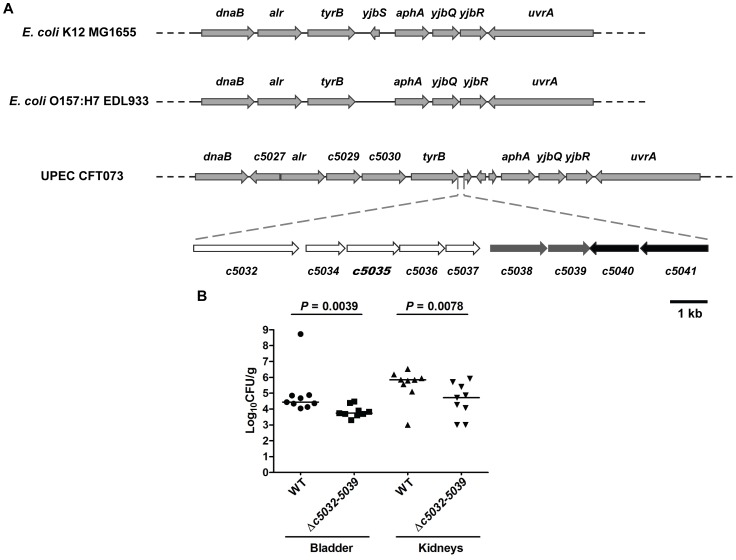
*c5032*-*5039* genes are located on a genomic island and contribute to UPEC fitness *in vivo*. (A) Similar genomic regions of *E. coli* K12 strain MG1655, EHEC strain EDL933, and UPEC strain CFT073 were compared and it was found that *c5032* to *c5041* genes are inserted between *tyrB* and *aphA* genes and can be considered as a genomic island. Within the island, three predicted operons shown as white, grey, and black arrows, respectively. Picture was drawn to scale. UPEC, uropathogenic *E. coli*; EHEC, enterohemorrhagic *E. coli*. (B) Island genes *c5032*-*5039* contribute to UPEC fitness *in vivo*. An *in vivo* competition assay was used. Equally mixed WT and Δ*c5032*-*5039*::Chl^r^ mutant bacteria containing approximately 2×10^9^ CFU were transurethrally inoculated into female mice. Two days after infection, the mice were sacrificed and their bladders and kidneys were aseptically removed. Wild-type and mutant bacteria were recovered by plating homogenized tissue samples on LB or LB containing chloramphenicol and their viable counts were determined. Each dot represents log_10_CFU/g in the bladder or kidney from an individual animal and the detection limit is 1000 CFU/g. Bars indicate the median log_10_CFU/g. A two-tailed Wilcoxon matched pairs test was performed, and the difference in colonization levels of WT and mutants was considered statistically significant if *P*<0.05.

Further analysis of genomic localization of *c5035* in CFT073 revealed that genes *c5032*-*5039* including *c5035*, together with TCS genes *c5041* and *c5040*, are encoded by a genomic island inserted between *E. coli* K12 genes *tyrB* and *aphA* (Fig. 4A). Not surprisingly, island genes *c5032-5039* were strongly associated with UPEC, and about 91.4% of UPEC strains tested containing *c5040*/*c5041* loci also possess *c5032*-*5039* genes (data not shown). Prediction by bioinformatics tools (http://linux1.softberry.com/berry.phtml and http://nostradamus.cs.rhul.ac.uk/~leo/sak_demo/) followed by reverse transcription PCR indicate that *c5032-5037* forms one operon and *c5038-5039* forms another (Fig. S2).

We then examined if this genomic island contributed to UEPC fitness *in vivo*. An *in vivo* competition assay was used to compare the colonization levels of Δ*c5032*-*5039* mutants with that of wild-type strain. Δ*c5032*-*5039* mutant was constructed by replacement with the *cat* cassette. Fig. 4B showed that there is significant reduction in colonization levels of Δ*c5032*-*5039* mutants in bladder and kidneys at 48 hours post-inoculation, as compared to the WT (*P*<0.05). These results clearly demonstrated that island genes *c5032*-*5039* are involved in UPEC colonization of murine urinary tract and it's tempting to presume that C5041/C5040 contributes to UPEC fitness through regulation of *c5032*-*5039* genes (The results that all other target genes in the genomic island are regulated by TCS C5041/C5040 were shown later in this paper).

### Island genes *c5032-5039* contribute to anaerobic α-KG utilization and α-KG induces their expression under anaerobic conditions

**Figure 5:**
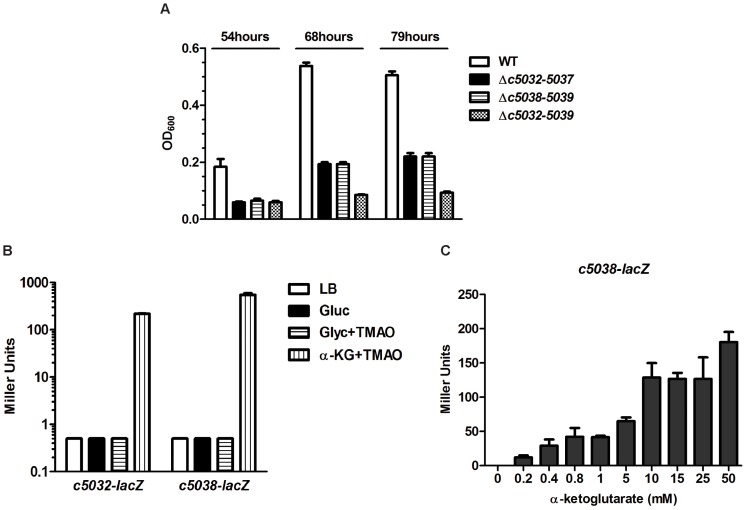
Contribution of *c5032*-*5039* to growth on α-KG and induction of *c5032*-*lacZ* or *c5038*-*lacZ* expression by α-KG. (A) *In vitro* growth of island gene mutants in M9 medium containing α-KG as the sole carbon and energy source. Optical density of the CFT073 WT and mutants during growth in M9 medium containing α-KG as the sole carbon source under anaerobic conditions was determined. Growth bars represent the average measurement at each time point from triplicate experiments. (B) Effects of different substrates on *c5032*-*lacZ* or *c5038*-*lacZ* expression. Gluc, glucose; Glyc, glycerol; TMAO, Trimethylamine *N*-oxide. (C) The induction of *c5038*-*lacZ* expression by α-KG is dose-dependent. Bacteria were grown in M9 medium containing 0.25% glycerol as the sole carbon source plus different amounts of α-KG as stimulus under anaerobic conditions.

The proteins encoded by *c5032*, *c5034*, and *c5035* are homologous to the E1, E2 and E3 components of α-KG dehydrogenase in *E. coli*, with similarity of 64%, 69%, and 55%, respectively; whereas, the proteins encoded by *c5036* and *c5037* are 70% and 83% similar to the beta- and alpha-subunits of succinyl-CoA synthetase from *E. coli*. α-KG dehydrogenase converts α-KG to succinyl-CoA, which can be further transformed to succinate by succinyl-CoA synthetase [43]. *c5038* is predicted to encode a putative dicarboxylate transporter with 13 transmembrane alpha-helices (TMHMM program [44]), showing 49% similarity to citrate/succinate antiporter CitT, and *c5039* encodes an enzyme belonging to malate/L-lactate dehydrogenase (Ldh_2) family. To test the hypothesis that these enzymes and the metabolic transporter are involved in α-KG utilization, we compared the growth of the WT, the Δ*c5032-5037* mutant, the Δ*c5038-5039* mutant, and the Δ*c5032-5039* mutant in M9 minimal medium with α-KG as the sole carbon source. Under aerobic conditions, no significant growth differences were observed among these strains (Fig. S3). However, when tested under anaerobic conditions, deletion of *c5032-5039* completely abolished the growth, and the Δ*c5032-5037* and Δ*c5038-5039* mutants also displayed statistically significant growth defects, as compared to the WT (Fig. 5A). We further tested their growth in M9 medium containing glucose, glycerol, or four-carbon dicarboxylate compounds, including succinate, fumarate, and malate, as the sole carbon source. In these cases, no differences were found among the strains tested (data not shown). These results indicated that genomic island genes *c5032-5039* specifically contribute to α-KG utilization under anaerobic conditions but play no or a very limited role in α-KG utilization under aerobic conditions.

The ability of α-KG to induce expression of *c5032*-*5039* was also examined. Chromosomal *c5032*-*lacZ* and *c5038*-*lacZ* transcriptional reporter fusions in CFT073*ΔlacZYA* were constructed in order to study the expression of operon *c5032-5037* and operon *c5038-5039*, and β-galactosidase activity in M9 medium using α-KG (40 mM) as the sole carbon source was also determined. Under anaerobic conditions, α-KG significantly induced the expression of *c5032* and *c5038*, as compared to the rich medium LB or M9 medium containing glycerol or glucose as the sole carbon source (Fig. 5B); while the analogs of α-KG such as 2-HO-glutarate, glutamate, glutarate, fumarate, or succinate could not. To verify that other target genes on the genomic island are also induced by α-KG, qRT-PCR was performed. RNA was isolated from bacteria grown in M9 medium (glycerol and TMAO) with or without inducer α-KG. As shown in Fig. S4, all of the genes tested were greatly induced in the presence of α-KG. We then examined the effect of α-KG concentration on the stimulation of *c5038* expression. Bacteria were grown in M9 medium plus glycerol and TMAO in the presence of various amounts of α-KG. We found that the induction of *c5038* by the presence of α-KG was dose-dependent with concentrations as low as 200 µM able to induce their expression under anaerobic conditions (Fig. 5C). These results indicate that island genes *c5032-5039* are induced by α-KG and involved in anaerobic utilization of α-KG.

### C5040 directly regulates the expression of *c5032-5039* and contributes to α-KG utilization under anaerobic conditions

**Figure 6:**
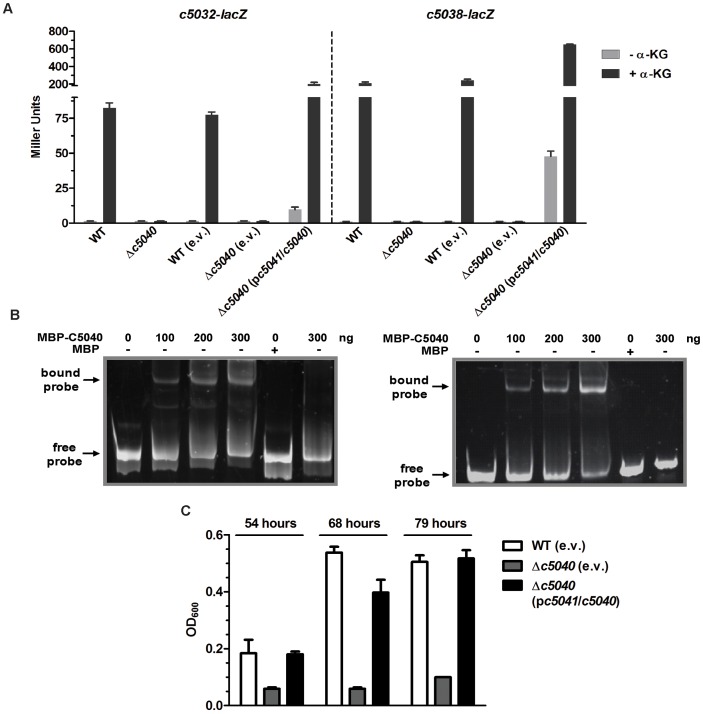
The role of *c5040*. (A) Effects of *c5040* on the expression of target genes in response to α-KG. Bacteria were anaerobically grown in M9 medium containing glycerol as sole carbon source and TMAO as electron acceptor in the absence or presence of α-KG. The expression of *c5032*-*lacZ* or *c5038*-*lacZ* in WT, Δ*c5040* (LMP107), and the complemented strains were represented by the β-galactosidase activity. (B) Non-radioactive EMSA studying the binding of MBP-C5040-His_6_ to the promoter regions. PCR products of *c5032* promoter region (left panel) and *c5038* promoter region (right panel) were used as probes. Purified MBP-C5040-His_6_ fusion protein was added in different concentration in each reaction mixture as indicated and MBP-His_6_ was used as negative control at 300 ng per each reaction. For the lane on the right on each panel, a negative control DNA fragment amplified from coding region of *c5036* was used. DNA fragments were stained with SYBR green. (C) *In vitro* growth of Δ*c5040* mutants. Optical density of the CFT073 WT, Δ*c5040* mutant, and the complemented strain during growth in M9 medium containing α-KG as the sole carbon source under anaerobic conditions was measured. Growth bars represent the average measurement at each time point from triplicate experiments.

To determine the regulatory role of C5040 in α-KG induction of target genes, chromosomal *c5032*-*lacZ* and *c5038*-*lacZ* transcriptional reporter fusions in the Δ*c5040* mutant were constructed. Strains were grown in M9 medium containing glycerol and TMAO in the presence or absence of inducer α-KG under anaerobic conditions. As shown in Fig. 6A, in wild-type genetic background, *c5032* and *c5038* expression was considerably induced in response to α-KG; in contrast, deletion of *c5040* abolished the expression of *c5032* and *c5038* to the background level under anaerobic conditions. Re-introduction of a plasmid construct p*c5041*/*c5040* carrying *c5041*/*c5040* genes with their native promoter into *c5040* mutants (LMP206 and LMP209) markedly increased expression of *c5032* and *c5038*; whereas, the empty plasmid vector did not affect the expression of *c5032* and *c5038*. In addition, the regulation of other target genes (*c5034*, *c5035*, *c5036*, *c5037*, and *c5039*) in the genomic island in response to α-KG by C5040 was confirmed using qRT-PCR (Fig. S4). Together, these results indicate that α-KG induction of *c5032*-*5039* is C5040-dependent (Fig. 6A).

To determine if C5040 directly regulates *c5032* and *c5038* expression, an electrophoretic mobility shift assay (EMSA) was performed. The promoter regions of *c5032* and *c5038* were predicted by BProm program (http://linux1.softberry.com) [45]. The potential binding sites for *c5032* and *c5038* were identified and found to be highly similar, with TGTGTG-N_13_-CGCGCA for *c5032* and TGTGCG-N_8_-CGCACA for *c5038*. DNA fragments containing the potential binding sites were then PCR amplified for use as probes (305 nucleotides in size for *c5032*, starting from −272 to +32 relative to translational start codon; 196 nucleotides in size for *c5038*, starting from −163 to +32 relative to translational start codon). Fragments amplified from the coding region of *c5036* were used as negative controls. Gene *c5040* encoding the RR was cloned into the expression vector pMal-c2x and fused with MBP-His_6_. MBP-C5040-His_6_ fusion protein retained its regulatory biological function since introduction of the plasmid construct expressing MBP-C5040-His_6_ fusion protein into Δ*c5040* mutant activated the expression of *c5038*; whereas, the empty plasmid did not affect expression of *c5038* (Fig. S5). As shown in Fig. 6B, the purified MBP-C5040-His_6_ fusion protein was able to shift the promoter fragments of both *c5032* and *c5038*, but not the control fragment. Meanwhile, use of the purified MBP-His_6_ fusion protein without C5040 was not associated with a detectable protein-DNA complex (Fig. 6B). These results demonstrate that C5040 directly binds to the promoters of the two operons.

To determine if C5040 is involved in the utilization of α-KG as the sole carbon source under anaerobic conditions, the growth properties of the WT, *Δc5040* mutants, and the complemented strains were compared. When grown in M9 medium with α-KG as the sole carbon source under anaerobic conditions, the *Δc5040* mutant strains showed a growth defect as compared to the WT. The *c5040*-complemented strain (Δ*c5040* carrying p*c5041*/*c5040*) significantly increased growth in this medium; while the empty plasmid vector (pGEN-MCS) did not improve growth of the mutant (*Δc5040*) (Fig. 6C).

### The role of C5041 in the expression of island genes *c5032-5039*

**Figure 7:**
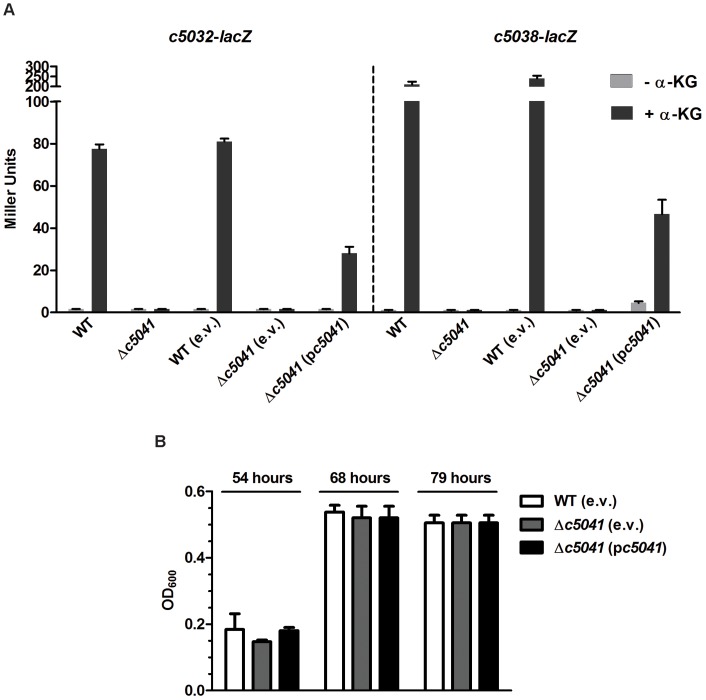
The role of *c5041*. (A) Effects of *c5041* on the expression of the target genes in response to α-KG. The expression of *c5032*-*lacZ* or *c5038*-*lacZ* in the WT, Δ*c5041* (LMP106), and the complemented strains were represented by the β-galactosidase activity. (B) *In vitro* growth of Δ*c5041* mutants. Optical density of CFT073 WT, Δ*c5041* mutant, and the complemented strain during growth in M9 medium containing α-KG as the sole carbon source under anaerobic conditions was measured. Growth bars represent the average measurement at each time point from triplicate experiments.

The regulatory role of HK C5041 was also determined. Chromosomal *c5032*-*lacZ* and *c5038*-*lacZ* transcriptional reporter fusions in the Δ*c5041* mutant (LMP106) were obtained to create LMP205 and LMP208, respectively. Strains were grown in M9 medium (supplemented with glycerol and TMAO) with or without inducer α-KG under anaerobic conditions. Deletion of *c5041* abolished the expression of *c5032* and *c5038* to background levels in the presence of α-KG. Re-introduction of the plasmid p*c5041* carrying the *c5041* gene with its native promoter into LMP205 and LMP208 mutants significantly increased their expression (P<0.05), indicating that C5041 positively affects expression of the target genes (Fig. 7A). This suggests that phosphorylation is very important for the regulatory function of RR C5040. In addition, the expression of other genes (*c5034*, *c5035*, *c5036*, *c5037*, and *c5039*) in the genomic island affected by C5041 was also confirmed using qRT-PCR (Fig. S4). The growth phenotypes of the *c5041* mutant and its complementation strain in difference medium were then tested. Interestingly, the growth of the WT and its derivative mutants (*c5041* mutant and complementation strain) in all media tested was statistically indistinguishable (Fig. 7B).

### Oxygen tension modulates expression of *c5032-5037* and *c5038-5039* directly via the TCS C5041/C5040

**Figure 8:**
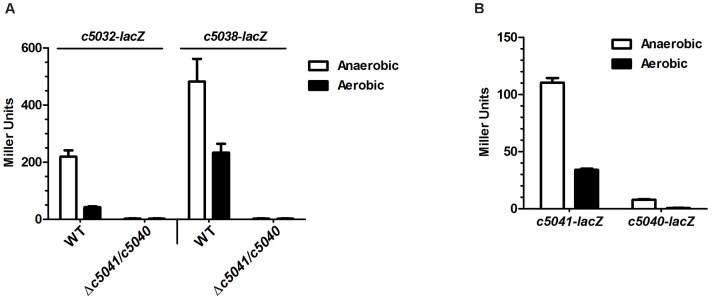
Oxygen tension modulates the expression of two operons in genomic island via TCS C5041/C5040. (A) Target genes' expression in *c5041*/*c5040* mutants under aerobic and anaerobic conditions. The expression of *c5032*-*lacZ* or *c5038*-*lacZ* in WT and Δ*c5041*/*c5040* mutant (LMP108) under aerobic and anaerobic conditions were compared. (B) Induction of *c5041*/*c5040* expression by oxygen deficiency. *c5041*-*lacZ* and *c5040*-*lacZ* transcriptional fusion strains were grown in M9 medium containing α-KG as the sole carbon source under aerobic and anaerobic conditions, respectively. β-galactosidase activity was measured and the values shown are means plus standard deviations of triplicate samples.

Island genes *c5032-5039* contribute to anaerobic utilization of α-KG but play a limited role under aerobic conditions causing us to suspect that oxygen might modulate expression of these genes. Indeed, the *c5032-5037* operon encoding a putative α-KG dehydrogenase and a putative succinyl-CoA synthetase was upregulated >5-fold in a reduced oxygen environment. Similarly, the expression of the *c5038-5039* operon, encoding a putative transporter of α-KG and a protein with unknown function, was upregulated 2-fold under anaerobic conditions, as compared to aerobic conditions (Fig. 8A).

To determine if oxygen regulates *c5032-5039* expression via C5041/C5040 but not through other independent pathways, the effects of *c5041*/*c5040* double deletion on the expression of *c5032-5039* were examined. Deletion of *c5041*/*c5040* completely abolished expression of the two operons under both aerobic and anaerobic conditions, supporting the hypothesis that oxygen regulates these two operons via TCS C5041/C5040 (Fig. 8A).

In addition, we wished to determine if oxygen deficiency regulates expression of the TCS itself. Under anaerobic conditions, the expression of *c5041* was about 3-fold greater than that observed under aerobic conditions (Fig. 8B), suggesting that *c5041* expression is significantly induced in response to anaerobiosis. Similarly, under anaerobic conditions, *c5040* expression is dramatically increased as compared to that under aerobic conditions (Fig. 8B). These results suggested that oxygen deficiency upregulated the expression of *c5041* and *c5040* and raised the possibility that oxygen modulates the expression of *c5032-5037* and *c5038-5039* operons via controlling *c5041*/*c5040* expression. However, these results did not rule out the possibility that anaerobiosis stimulates the typical TCS phosphotransfer reactions between C5041 and C5040, leading to increased DNA binding and up-regulation of target gene expression.

## Discussion

One of the greatest challenges confronted by all microorganisms is adapting to rapid changes of nutrient availability in different habitats. In the course of evolution, bacteria have developed several mechanisms to sense and utilize available nutrient sources associated with particular niches or to favor the most efficiently metabolizable nutrient sources when exposed to a range of choices. TCSs are major mechanisms enabling bacteria to couple environmental stimuli to adaptive responses [46]. The TCSs in *E. coli* K12 have been extensively studied [25]–[27]. Many TCSs that are common to both commensal and pathogenic *E. coli*, such as PhoQ/PhoP [31], QseC/QseB [29], [30], BarA/UvrY [28], and the AirS system [32] have been shown to contribute to virulence by mediating bacterial adaption to the host environment. However, no pathogen-associated TCS has yet been characterized in *E. coli*. Driven by the hypothesis that UPEC-associated TCSs exist to sense and respond to host environment signals distinct from those of intestinal *E. coli*, we identified a novel TCS C5041/C5040 significantly associated with UPEC strains, but not with EHEC, EPEC or ETEC strains. This TCS activated the expression of a genomic island involved in transport and metabolism of α-KG under anaerobic conditions. In view of these findings, C5041 was renamed KguS (α-ketoglutarate utilization sensor) and C5040 renamed KguR (α-ketoglutarate utilization regulator). These results indicated that UPEC might actively import α-KG and that α-KG could be an important carbon source for UPEC *in vivo*. Consistent with this observation, deletion of the TCS or its target island genes resulted in a significant reduction in a UPEC's colonization of the murine urinary tract. To our knowledge, this is the first report of a pathogen-associated TCS in *E. coli* that contributes to UPEC pathogenesis.

α-KG is an intermediate in the citric acid/tricarboxylic acid (TCA) cycle, which is a metabolic pivot for both catabolic and anabolic processes that supply key metabolic intermediates and energy in most eubacteria [47]. Many intermediates in the TCA cycle can be sensed by TCSs, which elicit an adaptive response in *E. coli*. Four-carbon (C_4_) dicarboxylate-sensing DcuS/DcuR was shown to control fumarate transportation (*dcuB*) and respiration (*frdABCD*) under anaerobic conditions, and under aerobic conditions affect the utilization of most of C_4_-dicarboxylates like succinate, fumarate, malate, and aspartate [48], [49]. In addition, tricarboxylate-sensing CitA/CitB was demonstrated to regulate anaerobic citrate fermentation genes *citCDEFXGT* encoding an active holo-citrate lyase catalyzing the breakdown of citrate to acetate and oxaloacetate and a citrate carrier [50]–[52]. This study has demonstrated that a novel TCS KguS/KguR responds to and only to α-KG, a five-carbon (C_5_) dicarboxylate by activating metabolic enzymes and transporter involved in anaerobic metabolism of α-KG. This is the first-time that such a TCS has been identified responding to C_5_-dicarboxylate in *E. coli*. Identification of the TCS regulating utilization of C_4_, C_5_-dicarboxylates and tricarboxylate suggested the tight control of the pathways involved in the metabolism of intermediates in *E. coli* TCA cycle.

As found in this study, α-KG can induce expression of the TCS target genes *c5032-5039*, while deletion of *kguS* or *kguR* abolished their expression in response to α-KG. By extrapolation from what we know of other TCSs and the results obtained in this study, it is very likely that α-KG serves as the signal molecule and HK KguS senses it via its periplasmic domain, then phosphorylates itself, and subsequently trans-phosphorylates KguR, which can then induce expression of the target genes. Very interestingly, overexpression of KguR can activate the expression of target genes independent of inducer α-KG. Similar observations have been reported for *Salmonella* where induction of target genes depends on the intracellular concentration of the response regulator PhoP and overexpression can substitute for phosphorylation and enhance the functionality of PhoP [53]. We further confirmed that oxygen tension modulated the expression of this pathway by regulating the expression of KguS/KguR itself. In *E. coli*, regulation by oxygen is usually mediated by the cytoplasmic regulator FNR and/or by TCS ArcB/A [54]. Currently, it is unknown if FNR and/or ArcB/A have any effect on the expression of KguS/KguR.

Genome sequencing has shown that the TCA cycle is a readily modified pathway in bacteria with many strains harboring variant TCA cycles. The common feature of these variants is the absence or repression of the α-KG dehydrogenase with the oxidative branch terminating in the production of α-KG and the reducing branch in succinate [55], [56]. Like any other *E. coli*, UPEC operate a complete TCA cycle aerobically, but the α-KG dehydrogenase shared by all *E. coli* is neither functional nor expressed under anaerobic conditions [47]. The KguS/KguR-controlled metabolic pathway in UPEC includes a putative α-KG dehydrogenase (*c5032-5035*) and a putative succinyl-CoA synthetase (*c5036-5037*), which were induced and contributed to utilization of α-KG under anaerobic conditions. To our knowledge, this is the first variant TCA cycle described in *E. coli* that links the oxidative and reducing branches under anaerobic conditions. To maintain carbon flux under oxygen-limiting or anaerobic conditions, many microaerophilic and obligate anaerobic organisms that lack α-KG dehydrogenase possess similar bypass pathways to metabolically link oxidative and reducing branches. *Helicobacter pylori* preferably growing under microaerophilic conditions use α-KG ferredoxin oxidoreductase to connect oxidative and reducing half-cycles [57]. Likewise, in *Mycobacterium* an α-KG decarboxylase structurally related to the E1 component of 2-oxoglutarate dehydrogenase can produce succinic semialdehyde (SSA), which is subsequently oxidized to succinate by succinic semialdehyde dehydrogenase (SSADH) [58]. This study and other groups' findings have highlighted the importance of the branched variants in adaptation to oxygen limiting or obligate anaerobic conditions and suggest that UPEC have different metabolic niches and needs compared to other *E. coli*.

The acquisition of additional metabolic traits often increases adaptability and competitiveness of pathogens in a new niche [18], [22]. This study found that TCS KguS/KguR and its controlled-anaerobic utilization pathway of α-KG were encoded by a genomic island in UPEC, which was absent from the genomes of commensal *E. coli* and intestinal pathogenic *E. coli*. These results were consistent with the previous report that *c5032* to *c5040* were among the 131 UPEC-specific genes identified using comparative genomic hybridization and *in silico* analysis of ten UPEC and four fecal/commensal isolates [59], and also suggested that this genomic island may facilitate UPEC's adaptation to *in vivo* colonization. This was confirmed by the observation that deletion of *kguS/kguR* resulted in a significant reduction in UPEC colonization in the bladders and kidneys of mice. However, deletion of *kguS/kguR* or its target genomic island genes did not affect growth in human urine *in vitro* under both aerobic and anaerobic conditions (data not shown). We reasoned that human urine might only be able to partially imitate the *in vivo* conditions of UPEC infection, whereas the contributions of KguS/KguR to UPEC pathogenesis may be restricted to some particular site of infection or pathogenic process, which cannot be represented by urine.

Interestingly, *Chlamydia trachomatis*, an obligate intracellular bacterial pathogen of urogenital infections [60], possesses an incomplete TCA cycle. Because of their small genome, chlamydiae lack many metabolic pathways retaining only the functions for performing key steps and interconversions of metabolites from the host cells. The fact that chlamydiae lack the first three enzymes of the TCA pathway and obtains α-KG from their host cell via a membrane transporter [56], [61] suggests that utilization of intracellular α-KG is important for the survival of this urogenital intracellular pathogen. It could be inferred from *Chlamydia* and hypothesized that α-KG might be one of the preferred carbon and energy sources for UPEC during infection. Previous studies supporting this hypothesis showed that the oxygen tension (PO2) in UPEC-infected tissue dropped to 0 mmHg within 3.5–4 h [62], suggesting UPEC encountered anaerobic conditions during infection. It was also reported that the concentration of α-KG in human cells and blood is about 10 µM, a concentration that was able to induce the expression of KguS/KguR-controlled pathway *in vitro* under anaerobic conditions in this study. The KguS/KguR-controlled transporter (C5038) differs from the constitutively expressed KgtP encoded by all *E. coli* [63], [64] and was induced under anaerobic conditions. During infection, this transporter allows UPEC to import α-KG from the infected tissue into the cell, and α-KG dehydrogenase and succinyl-CoA synthetase allow UPEC to take advantage of α-KG under anaerobic conditions, thus contributing to UPEC's *in vivo* fitness. More interestingly, proximal tubule cells in the kidney are able to accumulate α-KG. Proximal tubules are the primary site where organic anions (OA) of physiological, pharmacological, and toxicological importance are cleared from the body. Their clearance involves uptake of OA via OA-dicarboxylate exchange driven by α-KG gradients. Thus, intracellular concentration of α-KG in the proximal tubule cells is ∼100–400 µM, which is 10 to 40-fold higher than that found in any other cells [65]–[67]. UPEC undergo an intracellular lifestyle where they form biofilm-like communities [68], and UPEC are able to infect proximal tubule cells [62], [69]–[71]. However, very little is known about the metabolism of UPEC within these host cells. It is very likely that KguS/KguR-controlled anaerobic utilization pathway of α-KG would increase UPEC fitnessduring infections of renal proximal tubule cells. Future studies in our laboratory should help clarify these questions.

**Figure 9:**
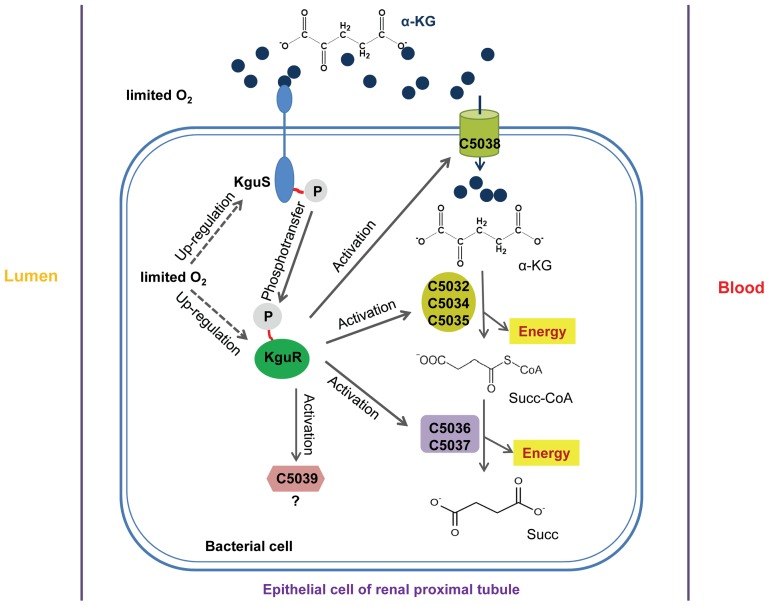
A schematic model for the regulatory mechanism of C5041/C5040 and its controlled target genes. In certain host environments of UPEC, such as the epithelial cells of renal proximal tubule, where oxygen tension is low and α-KG is abundant, KguS/KguR can sense and respond to these stimuli by activating the expression of *c5038* encoding dicarboxylate transporter and *c5032*-*5037* genes encoding metabolic enzymes, involved in the utilization of α-KG as carbon and energy source. Possession and functionality of this system may meet special metabolic needs for UPEC during urinary tract infections. Question mark indicates function is unknown. Blue dots, α-KG (α-ketoglutarate); Succ-CoA, succinyl-CoA; Succ, succinate.

In summary, our findings suggested a model that describes a novel regulatory and metabolic pathway in UPEC. For optimal growth during infection, HK KguS may sense α-KG, an abundant metabolite in UPEC infection site-renal proximal tubules, and then phosphorylate the RR KguR, which finally activates the import and utilization of α-KG (Fig. 9). These findings provide compelling evidence that this first UPEC-associated TCS enables *E. coli* to sense infection niche-specific stimuli and adapt to local nutrient availability, thus increasing its *in vivo* fitness. Hopefully, this and future studies on this regulatory system and metabolism pathway could lead to a better understanding of correlations between bacterial metabolism and virulence, but also the molecular pathogenesis of UPEC.

## Materials and Methods

### Ethics statement

7All animal procedures were conducted in accordance with NIH guidelines, the Animal Welfare Act and US federal law. The experimental protocol (Protocol number 4-11-7111-Z) for handling animals was approved by Institutional Animal Care and Use Committee at Iowa State University (IACUC). All surgery was performed under isoflurane anesthesia, and all efforts were made to minimize suffering.

The study using human urine was approved by Institutional Review Board (IRB ID: 04-171). Written informed consent was obtained from human participants and/or their legal guardians for urine collection.

### Bacterial strains and culture conditions

Strains and plasmids used in this study are listed in Table S1. Aerobic growth was achieved by shaking in air at 180 rpm and anaerobic growth by incubating in a Bactron chamber (Sheldon Manufacturing, Inc., OR) filled with gas mixture (N_2_, 90%; CO_2_, 5%; H_2_, 5%). For genetic manipulations, all *E. coli* strains were grown routinely in lysogenic broth (LB) medium. For growth and gene expression studies, bacteria were generally grown aerobically or anaerobically in M9 minimal salts with certain carbon sources indicated, supplemented with 2 mM MgSO_4_, 0.1 mM CaCl_2_, and 1 mg/ml vitamin B1. When used, trimethylamine N oxide (TMAO) and di- or tri-carboxylates were present at 40 mM. Glucose (0.5% v/v) or glycerol (0.25% v/v) was added as energy substrates, as indicated. Fresh mid-stream human urine was collected from six male and female consenting donors, pooled, and filter sterilized. Selective antibiotics and IPTG were added when necessary at the following concentrations: ampicillin (Amp), 100 µg ml^−1^; kanamycin (Kan), 50 µg ml^−1^;chloramphenicol (Chl), 25 µg ml^−1^; IPTG, 1 mM.

### Recombinant DNA techniques

Polymerase chain reaction (PCR), DNA ligation, electroporation and DNA gel electrophoresis were performed according to Sambrook and Russel [72] unless otherwise indicated. All oligonucleotide primers were purchased from Integrated DNA Technologies (Iowa) and are listed in Table S2. All restriction and DNA-modifying enzymes were purchased from New England Biolabs and used based on the suppliers' recommendations. Recombinant plasmids, PCR products, and restriction fragments were purified using QIAquick PCR purification kit or MinElute gel extraction kit (Qiagen, CA) as recommended by the supplier. DNA sequencing was performed at the DNA facility, Iowa State University. DNA and amino acid sequence analyses were performed using CloneManager software (Scientific & Educational Software, NC) and the search engine (http://blast.ncbi.nlm.nih.gov/Blast.cgi.) was used to identify conserved domain structures of the two-component system.

Deletion mutants were constructed using the lambda red recombinase system described by Datsenko and Wanner [73]. Chromosomal transcriptional *lacZ* fusion was constructed by homologous recombination of the suicidal plasmid pVIK112 carrying a fragment of complete 5′-region, 3′- region, or internal fragment of the target gene [74]. Briefly, PCR fragments of target genes were cloned into pVIK112 using *Eco*RI and *Xba*I sites. The resultant pVIK112 derivatives were introduced into CFT073Δ*lacZYA*::Chl^r^ by conjugation and the integration was allowed to occur. Conjugants were selected and confirmed by PCR. Chl^r^ was removed by transforming pCP20 plasmid carrying flippase [75]. For complementation, the coding sequences of genes plus their putative promoter regions were amplified from the CFT073 genome and independently cloned into pGEN-MCS [39] using *Eco*RI and *Sal*I restriction sites.

To construct the plasmid overproducing MBP-KguR-His_6_ fusion protein, a 1.4-kb fragment containing the coding region of *kguR* was obtained by PCR from genomic DNA using MalE/c5040Histag-F and MalE/c5040Histag-R carrying codons for 6×His and subsequently cloned into pMAL-c2x (New England Biolabs) using *Bam*HI and *Hind*III sites. The resultant plasmid contains MBP-KguR-His_6_ under the control of the P_tac_ promoter. The construction of control vector pMAL-MCS was achieved by replacing the MalE coding sequence with the MCS fragment from pEGFP plamid using *Nde*I and *Eco*RI sites.

### PCR genotyping

The nucleotide sequences publicly available of *c5041*/*c5040* genes were aligned by using the ClustalW2 program. The primers were selected from a relatively conserved region and on the basis of G/C content, annealing temperature and size of the amplicon. Multiplex PCR was carried out according to Johnson et al. [76].

### β-Galactosidase assays

Overnight LB cultures of *E. coli* containing the gene of interest-*lacZ* fusions were washed with PBS once and then were diluted 1∶100 in LB or M9 medium with the carbon sources indicated and grown at 37°C to log phase. These cultures were diluted 1∶10 in Z buffer and assayed for β-galactosidase activity using ortho-Nitrophenyl-β-galactoside (ONPG) as a substrate as described previously [77].

### 2D-DIGE

To prepare bacterial protein samples, isolated colonies were used to inoculate LB and cultures grown overnight at 37°C, the culture was further diluted 1∶50 to fresh LB medium and re-incubated; after OD 600 reached 1.0, 2 ml of culture (approximately 10^9^ CFU) was pelleted and the supernatant removed. Bacterial pellets were re-suspended in 20 ml of human urine and grown aerobically for 4 hours at 37°C. Bacteria grown in human urine were then collected by centrifugation and washed 3 times with sterile PBS. The resultant bacterial pellets were snap-frozen at −80°C and submitted to Applied Biomics, Inc. in California for Fluorescence difference in gel electrophoresis (2D-DIGE) [78].

### Reverse transcription PCR and quantitative real-time RT-PCR

RNA from *E. coli* CFT073 and its derivatives was stabilized by RNAprotect Bacterial Reagent (QIAGEN) and extracted using an RNeasy Mini Kit (QIAGEN) with a one-hour in-tube DNase digestion (QIAGEN) to remove possible DNA contamination according to the manufacturer's instructions. Three biological replicates of the each sample were prepared. The concentration of RNA was determined using a Spectrophotometer (ND-1000) (NanoDrop).

For the co-transcription test, one microgram of total RNA was reverse transcribed in triplicate using random hexamers and ImProm-II reverse transcriptase (Promega). Reactions without reverse transcriptase were used as a DNA contamination control. cDNA was then used as template for subsequent PCR reactions.

For quantitative real-time RT-PCR, melting curve analyses were performed after each reaction to ensure amplification specificity. Differences (n-fold) in transcripts were calculated using the relative comparison method, and amplification efficacies of each primer set were verified as described by Schmittgen et al [79]. RNA levels were normalized using the housekeeping gene *tus* encoding DNA replication terminus site-binding protein as an endogenous control [80]. Quantitative real-time RT-PCR (qRT-PCR) was performed with a Bio-Rad iQ5 iCycler detection system using iScript one-step RT-PCR kit with SYBR Green (Bio-Rad) according to the manufacture's instruction [81].

### Electrophoretic mobility shift assays

To study the binding of KguR to the DNA probe, electrophoretic mobility shift assays (EMSAs) were performed using the commercialized EMSA kit (Invitrogen, California) [82].

MBP-KguR-His_6_ fusion protein was purified to homogeneity using Ni-NTA Spin Columns and dialyzed against binding buffer. DNA probes were PCR amplified using specific primers and gel purified using a QIAGEN MinElute gel extraction kit. EMSAs were performed by adding increasing amounts of purified MBP-KguR-His_6_ fusion protein (0 to 300 ng) to the DNA probe (60 ng) in binding buffer (10 mM Tris (pH 7.5), 1 mM EDTA, 1 mM dithiothreitol, 50 mM KCl, 50 mM MgCl_2_, 1 µg ml^−1^ bovine serum albumin (NEB)) for 30 min at room temperature. Reaction mixtures were then subjected to electrophoresis on a 6% polyacrylamide gel in 0.5×TBE buffer (44.5 mM Tris, 44.5 mM boric acid, 1 mM EDTA, pH 8.0) at 200 V for 45 min. The gel was stained in 0.5×TBE buffer containing 1×SYBR Gold nucleic acid staining solution for 30 min.

### Experimental UTIs

Mouse infection studies were performed according to the methods of Johnson et al [83]. Female CBA/J mice (six to ten weeks of age) were anesthetized and inoculated via transurethral catheterization with a 20 µl (2×10^9^ CFU) challenge inocula per mouse. Overnight LB cultures for CFT073 and the mutant strain were pelleted and resuspended in sterile PBS, mixed in equal number and adjusted to make challenge inocula. To determine the initial CFU/mL, dilutions of each inoculum were plated onto LB plates with and without chloramphenicol. After 48 h, the mice were euthanized and the bladder and kidneys were aseptically removed, weighed, and homogenized in tubes containing PBS. Dilutions of the homogenized tissue were then plated onto duplicate LB plates with and without chloramphenicol or plates with different antibiotics to determine the bacterial concentration (CFU/g) of tissue. After overnight incubation, distinct colonies on plates were enumerated. The numbers of colonies on selective plates were subtracted from those on LB plates to obtain the number of wild-type bacteria. In the case of *in vivo* complementation studies, recovered bacteria were plated on LB plates with ampicillin and LB with ampicillin and chloramphenicol. Similarly, the numbers of colonies on ampicillin and chloramphenicol plates were subtracted from those on LB plates with ampicillin to obtain the number of wild-type bacteria. A group of 10 mice for each dual-strains challenge were used to determine alterations in fitness. The WT/Δ*c5041*/*c5041*::Chl^r^ competition assay was performed twice while other assays were performed once. For statistical analysis, a two-tailed Wilcoxon matched pairs test was used (Prism software, CA) and the threshold for statistical significance was a *P* value <0.05.

## Supporting Information

Figure S1 **Comparison of the proteomes from the WT, Δ** ***c5041*** **and Δ** ***c5040*** **mutants during growth in human urine.** Whole cell proteins of WT, Δ*c5041*::Chl^r^, and Δ*c5040*::Chl^r^ mutants cultured in urine were labeled with cy2, cy3, and cy5 respectively and analyzed by 2D-DIGE in a single gel. Red spots represent proteins induced in the mutant as compared to the WT; green spots indicate proteins repressed in the mutant as compared to the WT. Differentially expressed proteins were circled and numbered. (A) Comparison between the WT and Δ*c5041*::Chl^r^ mutants. Ten proteins were identified to be differentially expressed. (B) Comparison between the WT and Δ*c5040*::Chl^r^ mutants. Eleven proteins were identified to be differentially expressed.
(TIF)

Figure S2 **Operon formation represented by reverse transcription (RT)-PCR results using cDNA and a negative control without reverse transcriptase.** Primers were designed to span ORFs *c5032* & *c5034*, *c5034* & *c5035*, *c5035* & *c5036*, *c5036* & *c5037*, *c5037* & *c5038*, *c5038* & *c5039*, and *c5039 & c5040*. UPEC CFT073 were cultured in M9 medium containing glycerol as the sole carbon source. RNA was purified and reverse transcribed to cDNA. The RNA that was not reverse transcribed served as a negative control.
(TIF)

Figure S3 ***In vitro*** **growth of island gene mutants in M9 medium containing α-KG as the sole carbon and energy source under aerobic conditions.** Optical density of CFT073 WT and island gene mutants during growth in M9 medium containing α-KG as the sole carbon source under aerobic conditions was determined. Growth curves represent the average measurement at each time point from triplicate experiments.
(TIF)

Figure S4 **Effects of C5041 and C5040 on the induction of target genes on genomic island by α-KG.** Wild-type or mutant bacteria were anaerobically grown in M9 medium containing glycerol and TMAO in the absence or presence of α-KG. qRT-PCR expression values in the presence of α-KG are presented as relative values, as compared to that in the absence of α-KG. Error bars represent the standard deviations for 3 independent experiments.
(TIF)

Figure S5 **The induction of** ***c5038*** **expression by overproduced MalE/C5040 fusion protein.** The expression of *c5038*-*lacZ* in the WT transformed with the plasmid vector control (pMAL), pMAL-*c5040*, or pMAL-*malE*/*c5040* were represented by the β-Galactosidase activity. IPTG was present at 1 mM for induction of P_tac_ promoter.
(TIF)

Table S1 **Strains and plasmids.** The genotypes of all strains of *E. coli* utilized or constructed in this study and information about the plasmids used in this study.
(DOCX)

Table S2 **Oligonucleotides.** Oligonucleotide sequences used as PCR primers.
(DOCX)

Table S3 **Prevalence of** ***c5041*** **/** ***c5040*** **locus among** ***E. coli*** **isolates.**
(DOCX)
