# Supplementary figures and images for: A Novel Two-Component Signaling System Facilitates Uropathogenic Escherichia coli's Ability to Exploit Abundant Host Metabolites
Source: PLoS Pathog. 2013 Jun 27;9(6):e1003428. doi: 10.1371/journal.ppat.1003428 (PMC3694859; doi:10.1371/journal.ppat.1003428)

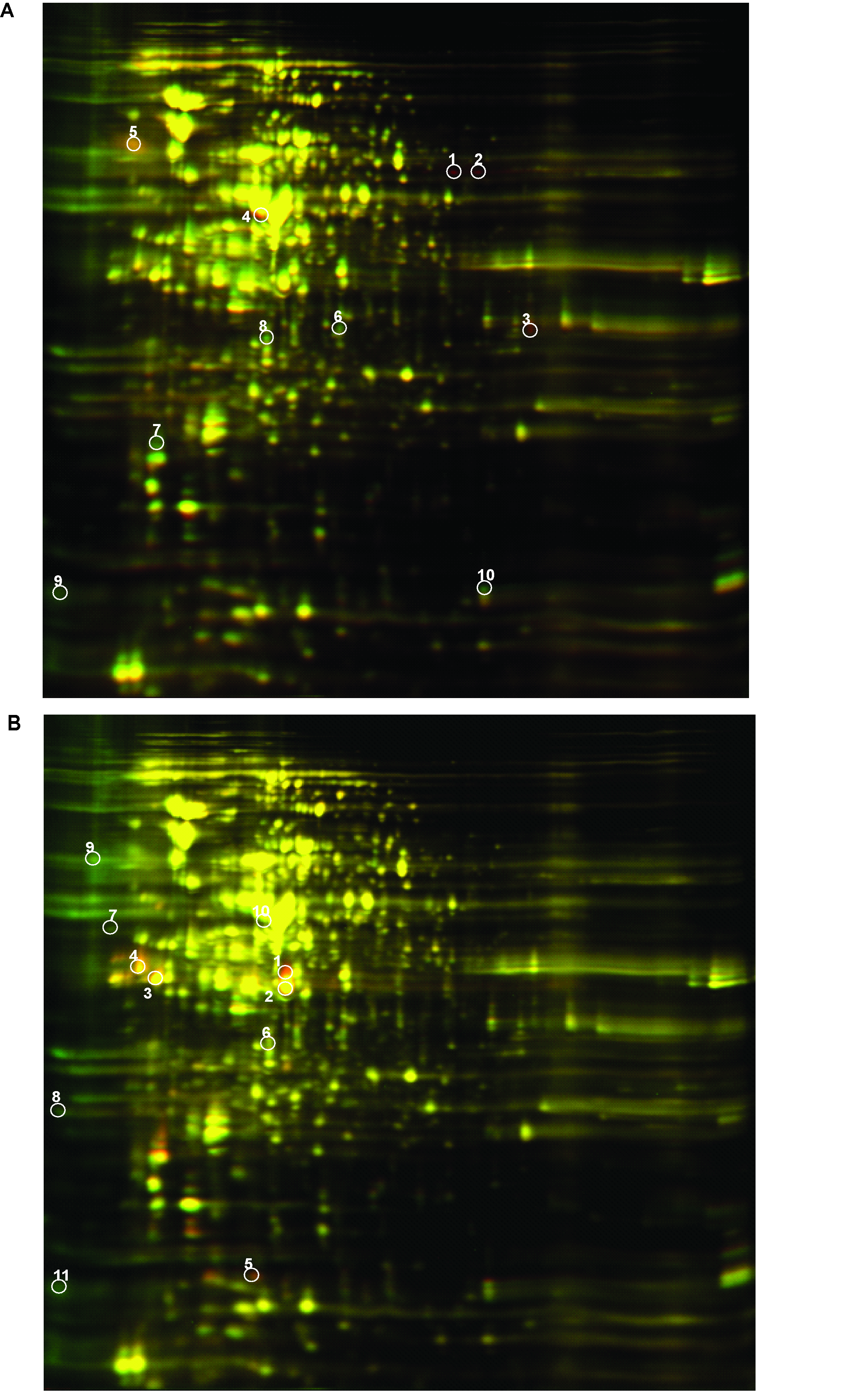

Supplement: Figure S1 — Comparison of the proteomes from the WT, Δ c5041 and Δ c5040 mutants during growth in human urine. Whole cell proteins of WT, Δc5041::Chlr, and Δc5040::Chlr mutants cultured in urine were labeled with cy2, cy3, and cy5 respectively and analyzed by 2D-DIGE in a single gel. Red spots represent proteins induced in the mutant as compared to the WT; green spots indicate proteins repressed in the mutant as compared to the WT. Differentially expressed proteins were circled and numbered. (A) Comparison between the WT and Δc5041::Chlr mutants. Ten proteins were identified to be differentially expressed. (B) Comparison between the WT and Δc5040::Chlr mutants. Eleven proteins were identified to be differentially expressed. (TIF) [file ppat.1003428.s001.tif]

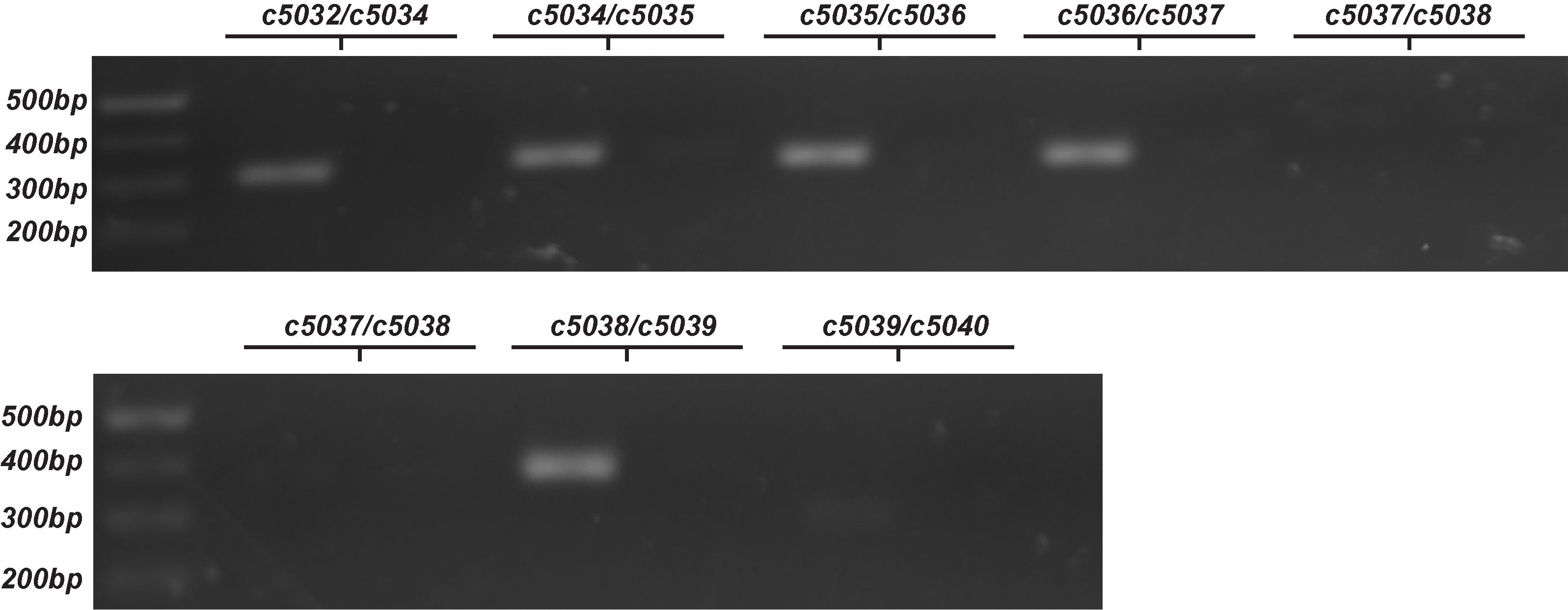

Supplement: Figure S2 — Operon formation represented by reverse transcription (RT)-PCR results using cDNA and a negative control without reverse transcriptase. Primers were designed to span ORFs c5032 & c5034, c5034 & c5035, c5035 & c5036, c5036 & c5037, c5037 & c5038, c5038 & c5039, and c5039 & c5040. UPEC CFT073 were cultured in M9 medium containing glycerol as the sole carbon source. RNA was purified and reverse transcribed to cDNA. The RNA that was not reverse transcribed served as a negative control. (TIF) [file ppat.1003428.s002.tif]

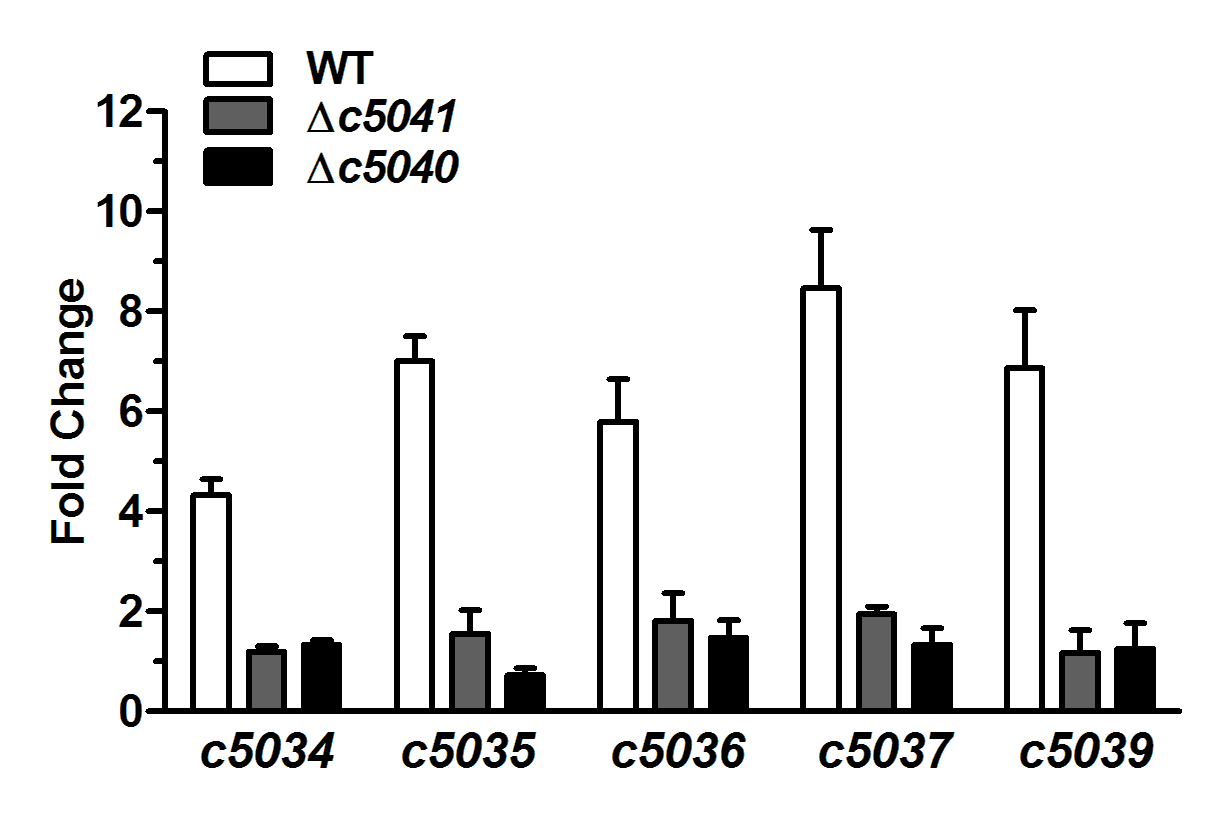

Supplement: Figure S4 — Effects of C5041 and C5040 on the induction of target genes on genomic island by α-KG. Wild-type or mutant bacteria were anaerobically grown in M9 medium containing glycerol and TMAO in the absence or presence of α-KG. qRT-PCR expression values in the presence of α-KG are presented as relative values, as compared to that in the absence of α-KG. Error bars represent the standard deviations for 3 independent experiments. (TIF) [file ppat.1003428.s004.tif]
