# Supplementary material for: A Novel Two-Component Signaling System Facilitates Uropathogenic Escherichia coli's Ability to Exploit Abundant Host Metabolites
Source: PLoS Pathog. 2013 Jun 27;9(6):e1003428. doi: 10.1371/journal.ppat.1003428 (PMC3694859; doi:10.1371/journal.ppat.1003428)
Supplement: Table S1 — Strains and plasmids. The genotypes of all strains of E. coli utilized or constructed in this study and information about the plasmids used in this study. (DOCX) [file ppat.1003428.s006.docx]

**Table S1. Strains and plasmids.** The genotypes of all strains of *E. coli* utilized or constructed in this study and information about the plasmids used in this study.

| **Bacterial strains and plasmids** | **Genotype or relevant characteristics** | **Source or Reference** |
| --- | --- | --- |
| ***Bacterial strains*** |  |  |
| *E. coli* DH5α | Plasmid propagation strain | Invitrogen |
| *E. coli* S17-λpir | RK2 *tra* regulon, *pir*, host for *pir-*dependent plasmids | [[1](#_ENREF_1)] |
| UPEC CFT073 | Blood isolate from a patient with acute pyelonephritis | [[2](#_ENREF_2)] |
| LMP10 | CFT073 ∆*lacZYA* | This study |
| LMP11 | CFT073 ∆*lacZYA*::Chl^r^ | This study |
| LMP100 | CFT073 ∆*c5041* | This study |
| LMP100Chl | CFT073 ∆*c5041*::Chl^r^ | This study |
| LMP101 | CFT073 ∆*c5040* | This study |
| LMP101Chl | CFT073 ∆*c5040*::Chl^r^ | This study |
| LMP102 | CFT073 ∆*c5041*/*c5040* | This study |
| LMP102chl | CFT073 ∆*c5041*/*c5040*::Chl^r^ | This study |
| LMP103 | CFT073 ∆*c5032* to *c5039* genes | This study |
| LMP104 | CFT073 ∆*c5032* to *c5037* genes | This study |
| LMP105 | CFT073 ∆*c5038*/*c5039* | This study |
| LMP106 | CFT073 ∆lacZYA ∆*c5041* | This study |
| LMP107 | CFT073 ∆lacZYA ∆*c5040* | This study |
| LMP108 | CFT073 ∆lacZYA ∆*c5041*/*c5040* | This study |
| LMP106Chl | CFT073 ∆lacZYA ∆*c5041*::Chl^r^ | This study |
| LMP107Chl | CFT073 ∆lacZYA ∆*c5040*::Chl^r^ | This study |
| LMP108Chl | CFT073 ∆lacZYA ∆*c5041*/*c5040*::Chl^r^ | This study |
| LMP203 | CFT073 ∆lacZYA *c5032*-*lacZ* | This study |
| LMP204 | CFT073 ∆lacZYA *c5038*-*lacZ* | This study |
| LMP205 | CFT073 ∆*lacZYA* ∆*c5041* *c5032*-*lacZ* | This study |
| LMP206 | CFT073 ∆*lacZYA* ∆*c5040* *c5032*-*lacZ* | This study |
| LMP207 | CFT073 ∆*lacZYA* ∆*c5041/c5040* *c5032*-*lacZ* | This study |
| LMP208 | CFT073 ∆*lacZYA* ∆*c5041* *c5038*-*lacZ* | This study |
| LMP209 | CFT073 ∆*lacZYA* ∆*c5040* c5038-*lacZ* | This study |
| LMP210 | CFT073 ∆*lacZYA* ∆*c5041/c5040* *c5038*-*lacZ* | This study |
| ***Plasmids*** |  |  |
| pMAL-c2X | expression vector | New England Biolabs |
| pMAL-MCS | *malE* was replaced by multiple cloning sites from pEGFP plasmid | This study |
| pMAL-c5040 | pMAL-c2x carrying *c5040* under the control of Ptac | This study |
| pMAL-MBP/c5040 | pMAL-c2x carrying MBP-C5040-6×His-tag under the control of Ptac | This study |
| pVIK112 | suicide plasmid for chromosomal *lacZ* transcriptional fusion | [[3](#_ENREF_3)] |
| pGEN-MCS | low copy plasmid for complementation | [[4](#_ENREF_4)] |
| p*c5041* | pGEN-MCS carrying *c5041* coding region and 500bp upstream promoter region | This study |
| p*c5041*/*c5040* | pGEN-MCS carrying *c5041/c5040* coding region and 500bp upstream promoter region | This study |
| pKD3 | template for λ-Red Chl^r^ cassette | [[5](#_ENREF_5)] |
| pKD4 | template for λ-Red Kan^r^ cassette | [[5](#_ENREF_5)] |
| pCP20 | encodes FLP recombinase for removal of resistance cassette | [[5](#_ENREF_5)] |
| pKD46 | λ-Red recombinase expression | [[5](#_ENREF_5)] |

1. Simon R, Priefer U, Puhler A (1983) A Broad Host Range Mobilization System for In Vivo Genetic Engineering: Transposon Mutagenesis in Gram Negative Bacteria. Nat Biotech 1: 784-791.

2. Welch RA, Burland V, Plunkett G, 3rd, Redford P, Roesch P, et al. (2002) Extensive mosaic structure revealed by the complete genome sequence of uropathogenic *Escherichia coli*. Proc Natl Acad Sci U S A 99: 17020-17024.

3. Kalogeraki VS, Winans SC (1997) Suicide plasmids containing promoterless reporter genes can simultaneously disrupt and create fusions to target genes of diverse bacteria. Gene 188: 69-75.

4. Lane MC, Alteri CJ, Smith SN, Mobley HL (2007) Expression of flagella is coincident with uropathogenic Escherichia coli ascension to the upper urinary tract. Proc Natl Acad Sci U S A 104: 16669-16674.

5. Datsenko KA, Wanner BL (2000) One-step inactivation of chromosomal genes in Escherichia coli K-12 using PCR products. Proc Natl Acad Sci U S A 97: 6640-6645.
