# Supplementary material for: A Novel Two-Component Signaling System Facilitates Uropathogenic Escherichia coli's Ability to Exploit Abundant Host Metabolites
Source: PLoS Pathog. 2013 Jun 27;9(6):e1003428. doi: 10.1371/journal.ppat.1003428 (PMC3694859; doi:10.1371/journal.ppat.1003428)
Supplement: Table S2 — Oligonucleotides. Oligonucleotide sequences used as PCR primers. (DOCX) [file ppat.1003428.s007.docx]

**Table S2. Oligonucleotides.** Oligonucleotide sequences used as PCR primers.

| **Primers** | **Sequence (5'-3')** |
| --- | --- |
| ***General PCR for cloning*** |  |
| pGEN-c5041-F | AGCTGAATTCCCGGACTGGAAAAAAGTACC |
| pGEN-c5041-R | ACTGGTCGACCGTACATCGTCACTATCCTC |
| pGEN-c5040/c5041-F | AGCTGAATTCCCGGACTGGAAAAAAGTACC |
| pGEN-c5040/c5041-R | ACTGGTCGACTTACGCCCGCAAAGGTATCG |
| pGEN-c5038/c5039-F | AGCTGAATTCTGATGCGCTGCGTTTATTCG |
| pGEN-c5038/c5039-R | ACTGGTCGACGCCGCGCAAGACTTTATATG |
| Ptac-c5040-F | ATCGCATATGAATAATAAATCCAGTGA |
| Ptac-c5040-R | ATCGGAATTCTTAGCTGGATGATTCTGGTC |
| MalE/c5040Histag-F | CTACGGATCCATGAATAATAAATCCAGTGA |
| MalE/c5040Histag-R | GCATAAGCTT*GTGGTGGTGGTGGTGGTG*GCTGGATGATTCTGGTCGAT |
| ***For lacZ fusion*** |  |
| c5032-lacZ-5'-F | TAGCGAATTCCTGGATTACCCGCTCTGGTG |
| c5032-lacZ-5'-R | GCTATCTAGATCGCGTTATCTCCACTAAGC |
| c5038-lacZ-5'-F | AGTCGAATTCTGGTGGTAATGCGGAAGAAC |
| c5038-lacZ-5'-R | ATCGTCTAGATATCGCCCAGTGGCAGAAGG |
| c5041-lacZ-5'-F | ATGCGAATTCTTCGCTTTCTGGCGAGAAGG |
| c5041-lacZ-5'-R | GCTGTCTAGAGAAACCGCGAGCATGATAAG |
| c5040-lacZ-3'-F | ATCGGAATTCTGTTATTGCAGCGACCAAGG |
| c5040-lacZ-3'-R | GCACTCTAGATTAGCTGGATGATTCTGGTC |
| ***For Deletion^a^*** |  |
| Del-c5041-F | GCGCCGCCTTCAGGATGATTACTGGCGTGGAGTGCGCCGTTCGAATTTCGgtgtaggctggagctgcttcga |
| Del-c5041-R | AAAATATCGTGGAATTATATATTTAAGAACAAATGGCGATTTCACATTACcatatgaatatcctccttag |
| Del-c5040-F | AAGGTATCGATTTGGGGTTACATCCTCAGGAATCAGCATTAGCTGGATGAgtgtaggctggagctgcttcga |
| Del-c5040-R | GCGCAGTAATGACATTAACCTGGCCTGAATGGGGAGAAGAACATGAATAAcatatgaatatcctccttag |
| Del-c5040/c5041-F | ACGCCCGCAAAGGTATCGATTTGGGGTTACATCCTCAGGAATCAGCATTAgtgtaggctggagctgcttcga |
| Del-c5040/c5041-R | CAGGGAATATACTGATGATAAATATAAATGCATTCAATGGATGATGAATGcatatgaatatcctccttag |
| Del-(c5032-c5039)-F | GCCATCCCCTACTATTCTGTCCCTTTTTTCGCCCACTTTTACATCATCTCgtgtaggctggagctgcttcga |
| Del-(c5032-c5039)-R | ATCGATACCTTTGCGGGCGTAATGTTCAACTACCCAGGACCAGAATGATAcatatgaatatcctccttag |
| Del-(c5032-c5037)-F | GCCATCCCCTACTATTCTGTCCCTTTTTTCGCCCACTTTTACATCATCTCgtgtaggctggagctgcttcga |
| Del-(c5032-c5037)-R | GGAGCGATATGCTCCCTTGATATTCATTTATGGATTACTTAATAACATTC*catatgaatatcctccttag* |
| Del-c5038/c5039-F | TATCCCATGGCGCACCACAAAAGCGTCGAATTATCCCTAATCCCGGCCTGgtgtaggctggagctgcttcga |
| Del-c5038/c5039-R | GATTCCTGAGGATGTAACCCCAAATCGATACCTTTGCGGGCGTAATGTTCcatatgaatatcctccttag |
| ***For EMSA*** |  |
| Prom5032-For | TTAATCAGCGCGGCATGTTC |
| Prom5032-Rev | CCGTACCAACAGTTAAAAAC |
| Prom5038-For1 | ATCTGTGTGGTAAGAGAATC |
| Prom5038-Rev1 | TGGTGCGCCATGGGATATTG |
| Prom5038-For2 | ATTATTTCTGGCGGTAGCGG |
| Prom5038-Rev2 | TCCGCACAGTAAATAAAAGG |
| Prom5038-For3 | CTGGGACTTGGGCAATCCAC |
| Prom5038-Rev3 | TCGAACGCCCGGAATTTATC |
| ***For Multiplex PCR genotyping*** |  |
| MultiP PCR-C5041F | AGCATTTGCCTCGCGCCATC |
| MultiP PCR-C5041R | GATTACTGGCGTGGAGTGCG |
| MultiP PCR-C5040F | TTAAACTGTGCAGGCTTACC |
| MultiP PCR-C5040R | CTGGTGGTGGGTCAGTAAAC |
| MultiP PCR-C5032F | ACAGGCTGCGGTAATACAAC |
| MultiP PCR-C5032R | TTTCACATCGCCTGAACCAC |
| MultiP PCR-C5033F | CACTCCTTACTGGCAGTATG |
| MultiP PCR-C5033R | CAGATTCCGGTAATACAGGG |
| MultiP PCR-C5034F | GCAGAGCGTGATGGATTTAC |
| MultiP PCR-C5034R | ATGCGATGGTCATAGCTGAG |
| MultiP PCR-C5035F | TCGAACGAAGTCCGTAAAGC |
| MultiP PCR-C5035R | ATTTGATCCGCAACCACCAC |
| MultiP PCR-C5036F | GTTTATTGTCAGCCCGGAAG |
| MultiP PCR-C5036R | ATTTCCAGCAGGGCGAAATC |
| MultiP PCR-C5037F | CAAACTGCCCAGGAGTTATC |
| MultiP PCR-C5037R | CCGGCAATATAACCTACGAC |
| MultiP PCR-C5038F | ATTACCGGGCTGGATAAACG |
| MultiP PCR-C5038R | GGTGGTTGGCCCTAATTCTG |
| MultiP PCR-C5039F | CCCGGAATGCTTAACAAGTG |
| MultiP PCR-C5039R | CGGTGAATTGATCAGGATCG |
| ***For qPCR and RT-PCR*** |  |
| tus-qPCR-F | CGATAACCTTTCGCAAGCAGCGTT |
| tus-qPCR-R | GGCAAATGACGATGCACCCATTCA |
| c5040-qPCR-F | CCGCCTGCATTGATGCTTTC |
| c5040-qPCR-R | TTGCGCGGCAGCTGTAATAG |
| c5032-qPCR-F | GTGGAGGAAATGGTCATTGG |
| c5032-qPCR-R | TTTCACATCGCCTGAACCAC |
| c5034-qPCR-F | GTCGGCCATTCTTGGGATGC |
| c5034-qPCR-R | ATGCGATGGTCATAGCTGAG |
| c5035-qPCR-F | TCTGTTTGCCGGTAACGGTC |
| c5035-qPCR-R | CAATCGCTCCGCCAAGTACG |
| c5036-qPCR-F | CTGAATGAGGCCCGTATCAC |
| c5036-qPCR-R | AGAGAATTGACGGCTTCCAC |
| c5037-qPCR-F | GAGCAGGCTATTGCGTATGG |
| c5037-qPCR-R | TCAGTTTGTCGCATCGCTTC |
| c5038-qPCR-F | CCTCACCATGTCGGTGATCC |
| c5038-qPCR-R | GGTGGTTGGCCCTAATTCTG |
| c5039-qPCR-F | CCCGGAATGCTTAACAAGTG |
| c5039-qPCR-R | GCGTTTTGCTTGTTCGATGG |
| Opr5032-5034-F | CACTCCTTACTGGCAGTATG |
| Opr5032-5034-R | GGGCCGGTATTTCCAGAATG |
| Opr5034-5035-F | AACGGCCAGGTTGTTATTGG |
| Opr5034-5035-R | CCGACATTGAGACACGTACC |
| Opr5035-5036-F | CGTACTTGGCGGAGCGATTG |
| Opr5035-5036-R | CATCTGCCAGTTGCGAAATC |
| Opr5036-5037-F | ATGAGGCCCGTATCACCCTG |
| Opr5036-5037-R | CTTACCTGGCGTCACTCCAC |
| Opr5037-5038-F | TTCCGGGCGTTCGATAAAGC |
| Opr5037-5038-R | CAATGATCGCCAACCACAGG |
| Opr5038-5039-F | CGGTCGGCATGACCATTCTG |
| Opr5038-5039-R | CCAGATATTTCGGTAGCAGC |
| Opr5039-5040-F | TATGCCAGAAGCTGCCAACC |
| Opr5039-5040-R | CGCCTGCATTGATGCTTTCG |

- Underlined are restriction cutting sites;
- Capital letters represent homologous fragments of the deleted genes.
