# Supplementary material for: A Novel Two-Component Signaling System Facilitates Uropathogenic Escherichia coli's Ability to Exploit Abundant Host Metabolites
Source: PLoS Pathog. 2013 Jun 27;9(6):e1003428. doi: 10.1371/journal.ppat.1003428 (PMC3694859; doi:10.1371/journal.ppat.1003428)
Supplement: Table S3 — Prevalence of c5041 / c5040 locus among E. coli isolates. (DOCX) [file ppat.1003428.s008.docx]

**Table S3. Prevalence of *c5041*/*c5040* locus among *E. coli* isolates.**

| ***E. coli* Strains** | **# of strains^a^** | **Prevalence** |
| --- | --- | --- |
| **Diarrheagenic *E. coli*** |  |  |
| **EPEC^b^** | **39** | **0%** |
| **EHEC^c^** | **66** | **0%** |
| **ETEC^d^** | **12** | **0%** |
| **Extraintestinal *E. coli*** |  |  |
| **UPEC^e^** | **200** | **70%** |

1. The presence of *c5041*/*c5040* in a well-characterized collection of 317 *E. coli* isolates of human origin was sought by multiplex PCR.
2. Enteropathogenic *E. coli* (EPEC)
3. Enterohaemorrhagic *E. coli* (EHEC)
4. Enterotoxic *E. coli* (ETEC)
5. Uropathogenic *E. coli* (UPEC).
